# Acute Kidney Injury in the Neonatal Period: Retrospective Data and Implications for Clinical Practice

**DOI:** 10.3390/children12070883

**Published:** 2025-07-03

**Authors:** Meidad Greenberg, Saray Sity-Harel, Sydney Benchetrit, Lewis Reisman, Tali Zitman-Gal, Daniel Erez, Maysam Shehab, Keren Cohen-Hagai

**Affiliations:** 1 Department of Pediatrics, Meir Medical Center, 59 Tchernichovsky St., Kfar Saba 4428164, Israel; 2 Department of Nephrology and Hypertension, Meir Medical Center, Kfar Saba 4428164, Israel; sydneybe58@gmail.com (S.B.); tali.gal@clalit.org.il (T.Z.-G.);; 3 Gray Faculty of Medical and Health Sciences, Tel Aviv University, Tel Aviv 6997801, Israel; daniel.erez@clalit.org.il (D.E.); shehabmaysam@gmail.com (M.S.); 4 Pediatric Nephrology Unit, Tel Aviv Sourasky Medical Center, Dana-Dwek Children’s Hospital, Tel Aviv 6423906, Israel; yehudareisman@gmail.com; 5 Department of Internal Medicine D, Meir Medical Center, Kfar Saba 4428164, Israel; 6 Department of Vascular Surgery, Meir Medical Center, Kfar Saba 4428164, Israel

**Keywords:** acute kidney injury, neonatal, chronic kidney disease

## Abstract

**Background**: Neonates, particularly those born prematurely or with low birth weight, face an elevated risk of developing Acute Kidney Injury (AKI) due to various factors. Perinatal and maternal considerations, often linked to preterm delivery, contribute to this heightened risk. **Methods**: A retrospective study of neonates admitted to the intensive care unit at a single Israeli Hospital who were diagnosed as having AKI. The study includes follow-up data on these children. **Results**: During the study period, 971 neonates were admitted to the Pediatric Intensive Care Unit (PICU), and 47 cases had a documented diagnosis of AKI. Thirty-four of them had available long-term data and were included in this analysis. A total of 13 out of 26 subjects with available blood pressure measurements had high blood pressure for their age percentile compatible with the definition of hypertension, and 6 out of 34 (17.6%) had proteinuria. **Conclusions**: These findings underscore the importance of increased clinical awareness and structured long-term follow-up for neonates who experience AKI.

## 1. Introduction

Neonates, particularly those born prematurely or with low birth weight, have an elevated risk of developing Acute Kidney Injury (AKI) due to various factors [1,2]. Perinatal and maternal considerations, often linked to preterm delivery, contribute to this heightened risk [3]. Given the vulnerability of neonates and the potential for long-term consequences associated with AKI in this population, it is a condition of significant importance.

Maternal comorbidities such as diabetes, chronic kidney disease (CKD), and hypertension, along with certain medications, may play a role in the occurrence of preterm delivery. Consequently, these neonates with immature kidneys also have reduced renal perfusion and oxygenation, rendering them susceptible to injury. The immature nature of their kidneys becomes a critical factor in the increased vulnerability to AKI. Furthermore, the prevalence of conditions such as perinatal asphyxia, sepsis, respiratory distress syndrome, and congenital heart disease is noteworthy among this at-risk neonatal population [3]. These additional factors contribute to the increased risk of AKI in neonates, emphasizing the significance of early recognition and comprehensive management in neonatal care [3].

AKI is defined as an increase in serum creatinine of 0·3 mg/dl or more (≥26.5 μmol/L) or 50% or more from the previous lowest value, or a urinary output of <1 mL/kg/h on postnatal days 2–7, according to the KDIGO workgroup AKI definition modified for neonates [2,4,5]. Several studies have investigated the prevalence of AKI in neonates, but the rates reported may differ based on the criteria used for diagnosis and the population studied. Incidence estimates have been reported to range from approximately 1.65% to 70%, depending on definition and population [1]. These rates can be influenced by factors such as gestational age, birth weight, the presence of underlying medical conditions, and exposure to nephrotoxic substances [1,2,6]. 

The prognosis of AKI in neonates is dependent on several factors, including the underlying cause, the severity of the injury, the gestational age of the infant, and the promptness of intervention. Neonates are a vulnerable population, and AKI in this age group is a serious concern that requires careful management. It is important to note that some neonates who appear to fully recover from AKI, with appropriate management, are at risk for developing hypertension, CKD, and other long-term complications due to reduced nephron mass [3].

While the short-term impact of AKI on neonatal prognosis has been extensively studied [1,2,3,4,6], the current analysis aimed to evaluate the long-term renal outcomes of neonates diagnosed with AKI during admission to a single-center pediatric intensive care unit (PICU) over a 20-year period.

## 2. Materials and Methods

### 2.1. Study Population

Meir Medical Center (MMC) is a 760-bed university hospital located in central Israel. It is the largest hospital in the district, which comprises a mostly urban population of Jews and Arabs. This retrospective study included all neonates born at MMC between 1 January 2000 and 1 January 2020, who were admitted to the PICU following live birth. The inclusion criteria were a documented diagnosis of AKI in the PICU discharge summary during the study period. Neonates without available medical records were excluded from the analysis. The data were collected through a retrospective review of the medical record of each patient discharged with a diagnosis of AKI.

### 2.2. Study Design

This study is a single-center retrospective study, which included all live births in MMC that were admitted to PICU and had the diagnosis of AKI documented either in their discharge summary or in the electronic medical record of their birth admission.

### 2.3. Variables and Measured Outcomes

The electronic medical records of the mother and newborn were reviewed, and the data, including maternal medical history, gestational age, birth weight, Apgar score, and medical treatment, were recorded. Data regarding kidney function, urinalysis, blood pressure, and kidney ultra-sonographic characteristics were also recorded for each individual.

All maternal comorbidities were recorded, including diabetes, hypertension, CKD, and others. Information on all chronic medications taken by the mother during pregnancy was also recorded.

Prematurity was defined as birth before 38 weeks of gestation, as commonly accepted. Standard criteria for PICU admission at MMC include birth before 34 weeks, birth weight under 2.5 kg, or perinatal complications such as asphyxia and conditions requiring admission to the PICU, including congenital anomalies.

Intrauterine Growth Restriction (IUGR) was defined as birth weight below the 10th percentile for gestational age, according to standardized fetal growth charts [7].

The diagnosis of AKI was based on documented diagnosis in the PICU discharge summary or in the electronic medical record of their birth admission. Each case was reassessed to confirm that it met the KDIGO criteria, specifically a rise in serum creatinine of at least 0.3 mg/dL (as previously described). Renal function tests were performed at the discretion of the treating physician and in response to clinical signs such as oliguria. According to the laboratory reference ranges, serum creatinine values below 0.3 mg/dL were considered normal for neonates. Blood pressure was obtained from the patients’ medical records.

Estimated glomerular filtration rate (eGFR) was estimated using the Schwartz formula, as described by Schwartz et al. [8], which is commonly used to assess kidney function in children with CKD. Ultrasound was performed by a specialist radiologist based on clinical judgment. The diagnosis of CKD during the follow-up period was based either on diagnostic coding in the medical record or on a documented eGFR of less than 60 mL/min/1.73 m^2^, when available.

### 2.4. Ethical Considerations

The study was approved by the local Ethics Committee (approval No. 0026-20-MMC, 2020). Due to the retrospective design of the study, the committee waved informed consent for patients included in this analysis.

### 2.5. Statistical Analysis

Data are expressed as mean ± standard deviation (SD), range [minimum to maximum] for continuous variables, and as absolute numbers or ratios (e.g., male/female) for categorical variables. Student’s *t*-test was used to compare continuous variables, and the chi-square test was used for categorical variables. *p*-values < 0.05 were considered statistically significant. All statistical analyses were performed using SPSS-27 (IBM, Armonk, NY, USA).

Results are reported according to the STROBE statement guidelines.

## 3. Results

**Figure 1:**
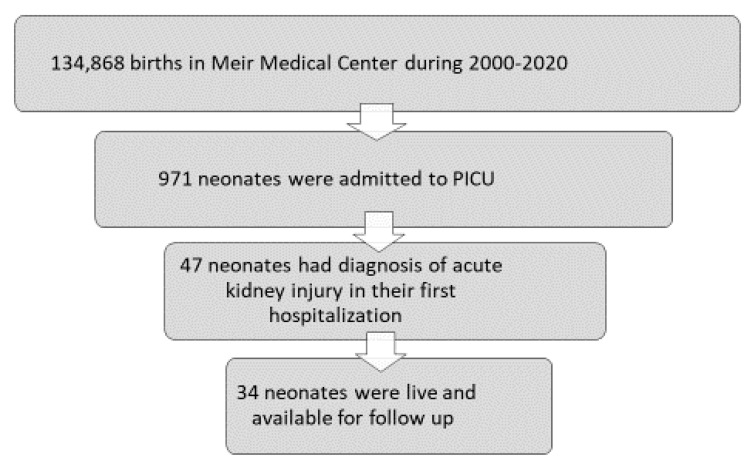
Flowchart of study participants.

During the study period, there were 134,868 births in the MMC (Figure 1). Among these, 971 neonates were admitted to the PICU and 47 cases had a documented diagnosis of AKI. A total of twelve neonates were excluded from the analysis due to early death occurring between 1 and 52 days after birth, and one additional was excluded due to unavailable data.

A total of 34 neonates were included in this analysis (Figure 1), with a mean postnatal follow-up of 6.3 ± 4.5 years. The mean birth weight of included children was 2473 ± 1024 g [range 600–4400], born at 35.2 ± 5.3 weeks [range 23–42 weeks]. Data are presented in Table 1 as mean ± SD.

### 3.1. Pregnancy

Among the neonates, the mothers were healthy in 27 cases (79.4%). Maternal comorbidities included diabetes in 3 cases (8.8%), hypothyroidism in 2 cases (5.8%), asthma in 1 case (2.9%), and celiac disease in 1 case (2.9%). Normal pregnancies were reported in 21 cases (61.7%), Table 1. Congenital anomalies of the kidney and urinary tract (CAKUT) were identified in three cases (8.8%), including one pelvic kidney, one case of polyhydramnios, and one case of hydronephrosis. Suspected cardiac malformations were noted in 2 cases (5.8%), echogenic bowel in 2 cases (5.8%), a single umbilical artery in 1 case (2.9%), and cleft lip and palate in 1 case (2.9%). Two neonates (5.8%) did not undergo prenatal evaluation, and IUGR was documented in 5 cases (14.7%).

The most common indications for delivery were term gestation or pathological monitoring (n = 18, 53%); other indications included pre-labor rupture of membranes (n = 5, 14.7%), preeclampsia (n = 3, 8.8%), placental abruption (n = 4, 11.8%), chorioamnionitis (n = 1, 2.9%), and oligohydramnios (n = 1, 2.9%).

### 3.2. AKI Hospitalization

More than half of the children developed oliguria during their hospitalizations (n = 20, 58.8%), with maximal serum creatinine levels of 2.3 ± 0.9 mg/dL, which decreased at discharge to 0.67 ± 0.37. Despite the evident kidney injury, only 50% of the children continued pediatric nephrologist follow-up.

### 3.3. Long-Term Outcomes

Sixteen children (47%) had no chronic diagnosis during follow-up. Others were diagnosed with developmental delay (n = 4, 11.8%), epilepsy (n = 3, 8.8%), ADHD (n = 3, 8.8%), cognitive impairment (n = 2, 5.9%), and cerebral palsy (n = 1, 2.9%).

During follow-up, four children were diagnosed with kidney disorders: dysplastic pelvic kidney (n = 1), nephrocalcinosis (n = 1, 2.9%) chronic kidney disease stage 3 (n = 1, 2.9%), and tubulopathy (Barter type 1; n = 1, 2.9%). We did not find a significant association between oliguria or hematuria during the PICU hospitalization and the subsequent development of hypertension, CKD, or structural kidney abnormalities during follow-up. However, we did observe a trend toward an association between perinatal asphyxia and the later development of CKD (0% vs. 21.4% in the asphyxia group, *p* = 0.088)

### 3.4. Blood Pressure

A total of 50% of the patients with available blood pressure measurements (13/26) had hypertension (high blood pressure for their age percentile) or above 140/90 as adults.

### 3.5. Proteinuria

**Figure 2:**
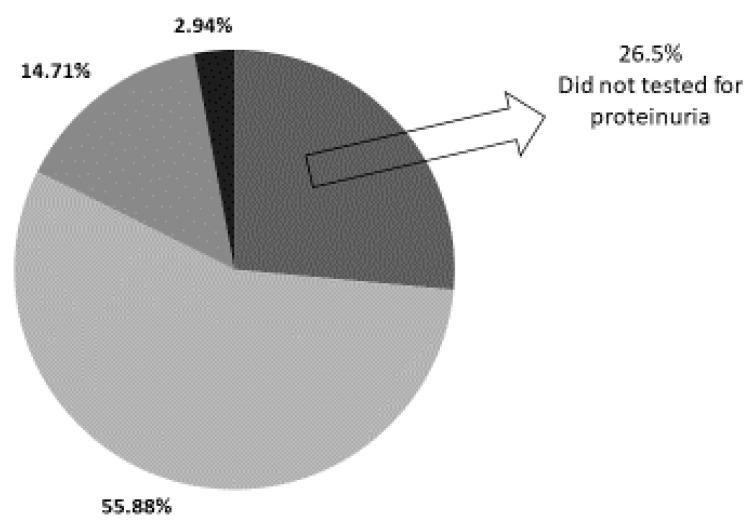
Prevalence of proteinuria in study cohort.

Six children (17.6%) had pathological proteinuria (five of them in the range of 30–100 mg/dL and one in the range of 100–300 mg/dL). Nine children were not tested for the presence of proteinuria (26.5%) and nineteen (55.88%) had no proteinuria on follow-up (Figure 2).

Out of 34 neonates evaluated after AKI, 6 children (17.6%) demonstrated pathological proteinuria on follow-up, 19 children (55.88%) had no detectable proteinuria, while proteinuria was not assessed in 9 children (26.5%).

### 3.6. Kidney Function

Thirteen cases (88.2%) had at least one available serum creatinine measurement following discharge from PICU.

A total of 3 out of 26 (11.5%) had CKD definition reflected by decreased estimated glomerular filtration rate (eGFR) (<60 mL/min/1.73 m^2^).

### 3.7. Sonographic Follow-Up of Kidneys

Pathological kidney findings on ultrasound (US), including signs of kidney disease such as hyperechogenic kidney parenchyma and corticomedullary differentiation, were observed in 7 cases (20.5%), while 11 cases (32.3%) had kidneys that were small for their age.

## 4. Discussion

This retrospective study evaluated long-term outcomes among neonates who developed AKI following admission to a single-center PICU over a 20-year period. Out of 134,868 live births during the study period, 971 newborns were admitted to the PICU, and 47 of them (4.8%) had a documented diagnosis of AKI. It is important to note that the true incidence of AKI may be underestimated, as early or transient episodes in neonates are often challenging to diagnose.

In our study, AKI was identified based on documentation in the hospital discharge letter. While the diagnostic criteria applied—a rise in serum creatinine and a reduction in urine output—are consistent with KDIGO guidelines [2,4], these measures may not be sufficiently sensitive for detecting AKI in neonates. Diagnostic accuracy in this population could be greatly enhanced through the use of novel biomarkers, such as urinary NGAL and plasma Cystatin C [2], which were not used in this retrospective study due to its design. Yet, the long-term consequences of AKI in this unique population warrant thorough evaluation due to their potential impact on kidney development, increased risk of chronic kidney disease, and impact on overall prognosis. This is especially important given the increasing survival of preterm infants, who may be particularly vulnerable to long-term renal complications.

In our cohort, neonatal AKI was associated with a substantial in-hospital mortality rate, with 13 out of 47 affected infants (27.7%) not surviving to discharge. Among the survivors, all appeared to have regained normal kidney function at the time of PICU discharge. However, accumulating evidence suggests that even apparently resolved episodes of AKI may leave residual kidney injury, especially among neonates with developing kidneys [9,10]. This subclinical damage can progress silently over time and eventually manifest as CKD, hypertension, or albuminuria—frequently as a result of focal segmental glomerulosclerosis [11]. These long-term complications highlight the significant prognostic impact of AKI in neonates and underscore the importance of structured follow-up for early detection and intervention. In our cohort, nine children were not tested for the presence of proteinuria (26.5%) despite their increased risk for CKD after they developed AKI. The mean birth weight of neonates with AKI in our cohort was 2473 g, with a mean gestational age of 35 weeks, indicating that the majority of infants were born prematurely with slightly lower than average birth weights for their gestational age. Additionally, three children (8.8%) were diagnosed with CAKUT.

Premature neonates and those with congenital anomalies or perinatal complications are predisposed to both acute and long-term dysfunction of several organ systems, including the pulmonary, neurological, and renal [12,13,14]. Recent studies have shown that kidney injury in this population may initially be subclinical, yet is associated with a significantly increased risk of developing hypertension and CKD later in life [11,12]. The high prevalence of hypertension, CKD, and imaging findings consistent with chronic kidney injury observed in our cohort aligns with these data and further underscores the importance of long-term renal follow-up in this vulnerable population.

The neonatal kidney, especially in premature infants, is particularly vulnerable to damage due to its underdevelopment. Kidney development continues late into pregnancy, with nephrons present by 36 weeks gestation, but not yet fully functional. The glomerular filtration rate (GFR) does not reach adult levels of 100 mL/min/1.73 m^2^ until around two years of age. Until that time, kidney function is still maturing, which places premature infants at an increased risk for renal complications. This underscores the heightened vulnerability of premature infants to kidney injury, as their kidneys are not yet fully capable of handling the demands of postnatal life [4,15].

Those newborns born before 36 weeks’ gestation are born with fewer nephrons. After delivery, there is a short period of continued nephron development, but this development is incomplete, and the resulting nephrons may not function properly [4,16].

Barry Brenner et al. [17] pointed out that humans and animals with fewer than the normal number of nephrons, either congenitally, or due to events later in life, are at risk for further kidney damage due to increased blood pressure and perfusion pressure in the remaining glomeruli. This is referred to as the ‘Hyperperfusion-Hyperfiltration model’. A child who is born with fewer nephrons, which includes every child born before 36 weeks’ gestation and every child born with CAKUT, is at risk for chronic kidney disease and hypertension later in life. In both pediatric and adult populations, AKI is increasingly recognized as a contributor to long-term renal impairment. This progression is often explained by the “multiple-hit” hypothesis of CKD, which suggests that an initial insult—such as CAKUT or prematurity—renders the kidneys more vulnerable to subsequent injuries. In this context, a second insult in the form of AKI can further compromise renal function, accelerating the path toward CKD.

Despite being discharged from the PICU with apparently complete recovery of renal function, within a few years, three (8.8%) of them were found to suffer from decreased eGFR, and 50% had from hypertension and/or proteinuria. As they get older, additional insults to the kidney, such as hypertension, atherosclerosis, obesity, and unhealthy lifestyle habits, e.g., tobacco, alcohol, and recreational drugs exposure, will presumably increase the risk of developing CKD and accelerating it [18]. This study was not designed to generate clinical guidelines, yet the findings of high rates of hypertension, proteinuria, and reduced GFR align with the recently published recommendations from the Neonatal Kidney Health Consensus Workshop [19]. Based on this broader expert consensus, preterm infants who experienced AKI should undergo structured, risk-based long-term nephrology follow-up. Specifically, the recommendations include a comprehensive kidney health evaluation at around six months after PICU discharge, and again at approximately two years of age for those at higher risk. Follow-up should include regular monitoring of blood pressure, serum creatinine and electrolytes, urinalysis (with assessment for proteinuria or albuminuria), and renal ultrasound. Additionally, families and healthcare providers should be educated about the potential long-term renal risks following neonatal AKI and the importance of ongoing surveillance.

This study has several limitations. First, it is a small, retrospective, single-center analysis, which may limit the generalizability of the findings. Second, the incidence of AKI may be underestimated due to underrecognition and challenges in diagnosing transient or subtle kidney injury in neonates, particularly in the absence of standardized diagnostic criteria, biomarker use, or routine testing. In our study, only about 5% of neonates admitted to the PICU were diagnosed with AKI, whereas most published studies report an estimated incidence of around 30% [3,20,21]. This discrepancy is likely due to our definition of AKI, which was based solely on a documented diagnosis in the discharge summary. In addition, this retrospective design relied on the diagnostic coding of AKI in the electronic medical records (EMRs), and milder cases were likely missed, as only more severe forms were probably documented. These factors limit the ability to accurately assess the true prevalence of AKI in this population. Third, there was no standardized timing for blood pressure measurements or laboratory assessments, and screening and follow-up for long-term kidney outcomes were not performed uniformly across the cohort. Some neonates did not receive structured nephrology follow-up, despite their increased risk, potentially leading to underdetection of late complications such as hypertension, proteinuria, reduced kidney function, and CKD. In this study, the diagnosis of CKD was based on diagnostic coding in the medical records rather than on the presence of microalbuminuria or decreased eGFR, as a substantial proportion of the children did not undergo routine microalbumin testing, and some were not followed by a nephrologist. Consequently, CKD is likely underdiagnosed, and the current retrospective study does not allow for an accurate estimation of its prevalence [22,23,24].

Although this is a retrospective, single-center study with a relatively limited sample size, it provides valuable insights and emphasizes several important clinical lessons. First, all neonates admitted to the intensive care unit should undergo thorough evaluation for AKI, given its significant implications for prognosis and long-term health outcomes [25]. Once AKI is identified, structured and long-term follow-up is essential. Early markers of kidney damage such as hyperfiltration, hypertension, or proteinuria, may be subtle or initially missed [26], yet they carry serious implications for future renal and overall health. This is especially important in light of the growing availability of effective therapies for CKD, which can potentially slow disease progression when initiated early in high-risk populations.

## 5. Conclusions

This retrospective single-center study suggests that neonatal AKI, although likely underrecognized, may be associated with substantial in-hospital mortality and potential long-term renal complications. These findings underscore the importance of increased clinical awareness, early identification, and structured long-term follow-up for neonates who experience AKI. Future prospective studies employing standardized diagnostic criteria and uniform follow-up protocols are needed to more accurately assess the true burden and long-term consequences of neonatal AKI.

## Figures and Tables

**Table 1:** Baseline characteristics of study cohort * (n = 34).

Variable	Absolute Number/Mean ± SD
Birth weight (grams)	2473 ± 1024
Birth week (gestational age)	35.2 ± 5.3
Male/female	15/19
Pregnancy	
Single/multiple	30/4
Normal/suspected malformation/IUGR	21/9/5
Delivery	
Normal/required respiratory intervention/required hemodynamic support	18/12/4
PICU hospitalization	
Aminoglycoside treatment	5 (14.7%)
Mechanical ventilation	16 (47.1%)
Vasopressor treatment	17 (50%)
Asphyxiation	16 (47.1%)
Complicated sepsis	3 (8.8%)
Oliguria	20 (58.8%)

* Data in Table 1 referred to data from birth as the baseline. Only available data are presented; missing values were not included in this table. Abbreviations: IUGR, Intrauterine Growth Restriction; PICU, Pediatric Intensive Care Unit.

## Data Availability

The original contributions presented in this study are included in the article. Further inquiries can be directed to the corresponding author.
